# Predicting Maximal Oxygen Uptake Using the 3-Minute All-Out Test in High-Intensity Functional Training Athletes

**DOI:** 10.3390/sports8120155

**Published:** 2020-11-30

**Authors:** Joshua D. Dexheimer, Shane J. Brinson, Robert W. Pettitt, E. Todd Schroeder, Brandon J. Sawyer, Edward Jo

**Affiliations:** 1 Department of Kinesiology, Azusa Pacific University, Azusa, CA 91702, USA; 2 Department of Kinesiology & Biology, Point Loma Nazarene University, San Diego, CA 92106, USA; sbrinson@pointloma.edu (S.J.B.); bsawyer@pointloma.edu (B.J.S.); 3 Department of Health Science, Rocky Mountain University of Health Professions, Provo, UT 84606, USA; rpettitt@rmuohp.edu; 4 Division of Biokinesiology & Physical Therapy, University of Southern California, Los Angeles, CA 90033, USA; eschroed@usc.edu; 5 Human Performance Research Laboratory, Department of Kinesiology and Health Promotion, California State University Pomona, Pomona, CA 91768, USA; ejo@cpp.edu

**Keywords:** VO_2max_, critical speed, D’, 3-minute all-out test, high-intensity functional training

## Abstract

Maximal oxygen uptake (VO_2max_) and critical speed (CS) are key fatigue-related measurements that demonstrate a relationship to one another and are indicative of athletic endurance performance. This is especially true for those that participate in competitive fitness events. However, the accessibility to a metabolic analyzer to accurately measure VO_2max_ is expensive and time intensive, whereas CS may be measured in the field using a 3 min all-out test (3MT). Therefore, the purpose of this study was to examine the relationship between VO_2max_ and CS in high-intensity functional training (HIFT) athletes. Twenty-five male and female (age: 27.6 ± 4.5 years; height: 174.5 ± 18.3 cm; weight: 77.4 ± 14.8 kg; body fat: 15.7 ± 6.5%) HIFT athletes performed a 3MT as well as a graded exercise test with 48 h between measurements. True VO_2max_ was determined using a square-wave supramaximal verification phase and CS was measured as the average speed of the last 30 s of the 3MT. A statistically significant and positive correlation was observed between relative VO_2max_ and CS values (r = 0.819, *p* < 0.001). Based on the significant correlation, a linear regression analysis was completed, including sex, in order to develop a VO_2max_ prediction equation (VO_2max_ (mL/kg/min) = 8.449(CS) + 4.387(F = 0, M = 1) + 14.683; standard error of the estimate = 3.34 mL/kg/min). Observed (47.71 ± 6.54 mL/kg/min) and predicted (47.71 ± 5.7 mL/kg/min) VO_2max_ values were compared using a dependent t-test and no significant difference was displayed between the observed and predicted values (*p* = 1.000). The typical error, coefficient of variation, and intraclass correlation coefficient were 2.26 mL/kg/min, 4.90%, and 0.864, respectively. The positive and significant relationship between VO_2max_ and CS suggests that the 3MT may be a practical alternative to predicting maximal oxygen uptake when time and access to a metabolic analyzer is limited.

## 1. Introduction

The use of physiological testing informs the sports performance coach and sports scientist about competitive athletic success as well as aids in development of endurance training programs by prescribing and monitoring training loads to elicit positive physiological adaptations. The applications of these measurements include the assessment of aerobic fitness, the prescription of exercise training workouts, and the prediction of endurance performance [1,2,3,4,5]. Maximal oxygen uptake (VO_2max_) [6] and other aerobic/anaerobic thresholds (i.e., lactate threshold (LT), maximal lactate steady state (MLSS), and their physiological equivalents) [4,7,8,9,10,11] are key indicators of endurance performance. However, traditional procedures and instrumentation to measure these variables are both time intensive and require expensive as well as sophisticated equipment. Laboratory measurements of VO_2max_ and aerobic/anaerobic thresholds may take anywhere from 5 to 26 min [12,13,14] consisting of several stages and require access to costly equipment such as a metabolic analyzer. Not to mention, the accuracy of aerobic/anaerobic thresholds are influenced by several intrinsic and extrinsic variables [15,16,17] as well as various methodological techniques [18,19]. This has led to the use of several submaximal and field tests, as well as other technology, to assess endurance performance.

Both submaximal laboratory-based tests as well as field tests provide a means to overcome the barriers and prohibitive costs associated with measuring endurance performance. For instance, several submaximal tests only require access to a treadmill or cycle ergometer and may be used to accurately estimate VO_2max_ in clinical [20], general [21], and athletic populations [22]. Further, field tests, performed outside of a laboratory setting, may be used to estimate both VO_2max_ [23] and anaerobic thresholds, like the MLSS [24,25]. It has also been proposed that wearable technology may be used for estimating VO_2max_ [26]. However, the accuracy of such devices still warrants further investigation [27]. Though these seem like feasible alternatives to the expensive laboratory measurements, the testing procedures are still time intensive and may require complex equations with multiple variables [28]. If time is a constraint, the Queen’s college step test is one of the most feasible and time-efficient tests, totaling only 3 min. However, it only provides an estimate of VO_2max_ [29]. Therefore, a short field test that could non-invasively assess both fractional and maximal threshold values with comparable accuracy to that of metabolic testing would be a valuable aid in optimizing training of recreational and competitive endurance athletes.

The 3 minute all-out test (3MT) may be the most cost and time-efficient means of assessing both fractional and maximal threshold values. The 3MT is short in duration and has demonstrated to be both a reliable and valid test for the measurement of critical speed (CS) and the finite capacity of running speeds above CS, D prime (D’) [30,31]. Critical speed has demonstrated a relationship to both fractional and maximal threshold values [18,32,33,34,35]. A relationship exists between CS and MLSS [32], although CS may be a more sensitive and reliable fractional threshold measurement of the upper limit of the heavy exercise intensity domain [18,33]. Evidence also suggests that CS and VO_2max_ are positively correlated, meaning that those with a higher VO_2max_ also have a greater CS value [34,35]. This relationship is further displayed, as VO_2max_ measured from incremental stage tests was similar to maximal values attained by traditional CS testing [36]. These findings are additionally substantiated by similar VO_2max_ values obtained from a graded exercise test (GXT) to both a traditional running and shuttle 3MT [37]. However, few studies have examined the ability of CS to predict VO_2max_ [34,38]. Moritani et al. [38] and Kendall et al. [34] have revealed that VO_2max_ may be derived from regression equations using the cycling the rowing equivalents of CS and D’ from traditional CS testing. Traditional CS testing consists of several exercise trial measurements, sometimes across multiple days, at various intensities or distances, which may take longer than a GXT to measure VO_2max_ [36,38,39,40]. Thus, the 3MT may provide a practical alternative to measuring both CS and VO_2max_ in a fraction of the time. 

To our knowledge, no other short duration field test (<3 min), requiring minimal equipment, provides both fractional and maximal threshold values as can be determined in a laboratory setting using a GXT or multi-stage test. Hence, the aim of this study was to determine the relationship between CS and D’ from a running 3MT and VO_2max_ from a GXT to develop a regression equation to predict relative VO_2max_ based on significant independent variables (CS and D’). The purpose was to determine whether the 3MT may be a practical testing alternative in identifying aerobic fitness within individuals when access to expensive instrumentation is restricted. It was hypothesized that there would be a significant relationship between CS and VO_2max_ as well as D’ and VO_2max_ when assessed from the 3MT and GXT, respectively. 

## 2. Materials and Methods

### 2.1. Participants 

Thirty-seven men and women were originally recruited from a local high-intensity functional training (HIFT) gym. Twelve participants were excluded from the final analysis due to invalid tests discussed below. Thus, final analysis was conducted on twenty-five recreational HIFT athletes. Participant demographic data was collected on the first visit and is displayed in Table 1. All participants provided written informed consent and the protocol under which this study was conducted was approved by the Point Loma Nazarene University Institutional Review Board (ID# 17204). Participants recruited were between the ages of 18 and 45, healthy and uninjured, and had at least 1 year of exercise training experience providing them the ability to perform standard HIFT workouts without scaling or modifications [41]. Normative data collected on two standardized workouts that participants completed in this study indicated both men and women to both be within approximately the 30th and 60th percentiles of competitive workout performance, representing a recreationally competitive group [42]. 

### 2.2. Protocol 

All testing procedures took place at Point Loma Nazarene University within the Exercise Physiology Lab and the Track and Field Stadium. Upon completion of descriptive assessments, participants completed a 3MT and GXT with a supramaximal square-wave verification phase (VP) on separate days with at least 48 h between each test to allow sufficient recovery time for the participants [43]. Participants were required to be 2–3 h fasted having had no caffeine prior to testing [44].

### 2.3. Body Composition and Anthropometrics 

Each participant underwent a series of measurements evaluating body composition and anthropometrics. Height was measured with participants standing barefoot on a stadiometer (Seca Inc. Hamburg, Germany). Air displacement plethysmograph via the BodPod (Cosmed, Concord, CA, USA) was used to assess body composition where body fat percentage was calculated using Siri’s formula [45]. The BodPod has demonstrated to be both a valid and reliable measurement of body composition and was calibrated daily before testing according to the manufacturer’s instructions [45,46].

### 2.4. Graded Exercise Test 

All participants completed an initial ramp GXT followed by a supramaximal square-wave VP. Due to high volume of testing, two Parvo Medics TrueOne 2400 (Parvo Medics, Sandy, UT, USA) metabolic analyzers were used to continuously collect gas exchange values, while heart rate was measured with a Polar heart rate monitor (Polar, Lake Success, NY, USA). Flowmeter and gas calibrations were performed prior to each test as per the manufacturer’s instructions. Previous findings have revealed low interunit errors of 1.5–2.1% [47] and utilizing two units allowed for two participants to be tested at the same time. If repeat tests were needed the same unit was used within a subject. After sampling resting data for 2 min, participants began a 5 min walking warm up between 3.0 and 3.5 mph. Upon completion of the warm up, the individualized custom treadmill GXT commenced where participants started at 5 mph with a 3% grade and each minute the speed increased with the grade remaining constant. The custom GXT protocol was created using a non-exercise regression equation to predict VO_2max_ and then deriving a speed estimate with that metabolic value [48]. The estimated peak was then divided by the number of stages to yield at GXT duration within 8–12 min [48]. Verbal encouragement was used as a form of extrinsic motivation to motivate participants to their maximum effort. The VO_2max_ obtained from the initial ramp phase was derived as the average of the two-highest consecutive 15 s oxygen uptake averages. Upon completion of a 10 min walking active recovery between 1.5 and 3.5 mph, each subject performed a supramaximal square-wave verification test at 105% of the speed, with the same grade, obtained during the initial ramp test [49,50,51]. The initial ramp GXT VO_2max_ and VP VO_2max_ had to be within 3% for the GXT VO_2max_ to be accepted as a true max [52]. If greater than 3%, participants came back to perform the test again on the same unit, or their results were excluded from the final analysis. 

Results from the GXT were used to identify the speed evoking gas exchange threshold (GET) and VO_2max_ using a linear interpolation method [53]. The GET was determined using the v-slope method [54]. The physiological response to a given change in speed during a GXT is not instantaneous, rather, it is delayed typically by 1 min. Hence, the speed evoking a specific gas exchange value is associated with the specific speed preceding the measurement by 1 min. To calculate this, speed (mph) equaled the incremental stage change value divided by four, as data was averaged every 15 s and stage speed increased each minute. These calculations were used to determine the average of the speeds at GET and VO_2max_ (50% Δ) to confirm CS results from the 3MT [30]. Calculating 50% Δ allowed us to screen for pacing during the 3MT as CP is the approximate mean value (50% Δ) for power evoking gas exchange threshold (GET) and VO_2max_, as determined from a GXT [31].

### 2.5. 3 Minute All-Out Test

The 3MT was conducted on a flat track following a general running warm up and standardized dynamic stretching routine created by a Certified Strength and Conditioning Specialist. Participants ran as fast as possible around the track for 3 min and 5 s as a GPS tracking app, Sports Tracker (Amer Sports Digital Services Oy, Vantaa, Finland), collected second-by-second speed data which was used to derive CS and D’. The running 3MT presumes that an athlete will expend D’ within 2.5 min of all-out effort, whereby the mean speed between 2.5 and 3.0 min will reach a nadir at CS [30]. Thus, D’ from a running 3MT was derived by Equation (1), where time (t) equals 150 s, S150 s (m/s) equals the average speed for the first 150 s, and CS (m/s) is the average speed between 150 s and 180 s [30].

D^′^ = t (S150s − CS) (1)

As previously mentioned, 50% Δ, calculated as the average of the speeds at GET and VO_2max_, was used to detect pacing during the 3MT. A CS considerably different from 50% Δ (≥3.5%) denoted an inaccurate and/or inflated CS due to pacing and warranted retesting or exclusion of that data. As the GXT protocol consisted of running at a constant 3% grade, a series of regression equations were utilized to convert treadmill speed and grade to equivalent outdoor running speed [55].

### 2.6. Statistical Analysis

Preliminary analyses were conducted to ensure no violation of the assumptions of performing a regression analysis. Cook’s distance and box plots were used to inspect outliers. The Kolmogorov–Smirnov Statistic and normal P–P plots were used to assess normality, linearity using scatterplots, and multicollinearity from values of tolerance and variance inflation factor. Lastly, homoscedasticity was evaluated using a scatterplot of standardized residuals and predicted values. Descriptive statistics were performed on participant characteristics. Data analyses were performed using SPSS Version 25. Pearson’s product moment correlation analyses were completed to determine the strength of the relationship between the CS, D’, and observed relative VO_2max_ values. A multiple stepwise linear regression was used to determine the relative influence of significant correlative data and sex, creating a prediction equation to determine relative VO_2max_. 

Relative consistency between tests was evaluated using intraclass correlation coefficients (ICC α) whereas absolute consistency was evaluated using coefficient of variation (CV%) and typical error (TE) [56]. Comparisons between observed and predicted VO_2max_ as well as 50% Δ and CS were made using paired *t* tests. The level of significance (α-level) for statistical analysis was set at *p* < 0.05. All data is reported as the mean ± standard deviation (SD). 

## 3. Results

Preliminary analyses revealed no violations, ensuring all assumptions were met for a regression analysis. However, as previously mentioned, twelve participants were excluded from the final analysis due to one of the following: inaccurate and/or inflated CS due to pacing on the 3MT (9 participants) or a VP greater than 3% (3 participants). Relative VO_2max_ (47.71 ± 6.54 mL/kg/min) and VO_2_ verification (47.18 ± 6.19 mL/kg/min) displayed internal consistency (CV% = 0.96, TE = 0.45 mL/kg/min). An average difference of only 1.0% between the ramp and verification protocols indicated participants reached a true VO_2max_ [49,52,57,58]. Lastly, no significant difference was detected between 50% Δ (3.59 ± 0.48 m/s) and CS (3.56 ± 0.55 m/s) (t = 0.524, *p* = 0.605). 

The Pearson’s product-moment correlation coefficient displayed a strong significant positive correlation between CS and VO_2max_ (r = 0.819, *p* < 0.001) but not between D’ and VO_2max_ (D’ = 208.5 ± 54.3 m; r = −0.198, *p* = 0.344). A stepwise linear regression was used to generate a prediction equation (Equation (2)) for determining relative VO_2max_ from CS and sex.

Relative VO_2max_ = 8.449(CS) + 4.387(F = 0, M = 1) + 14.683, SEE = 3.34 mL/kg/min (2)

**Figure 1:**
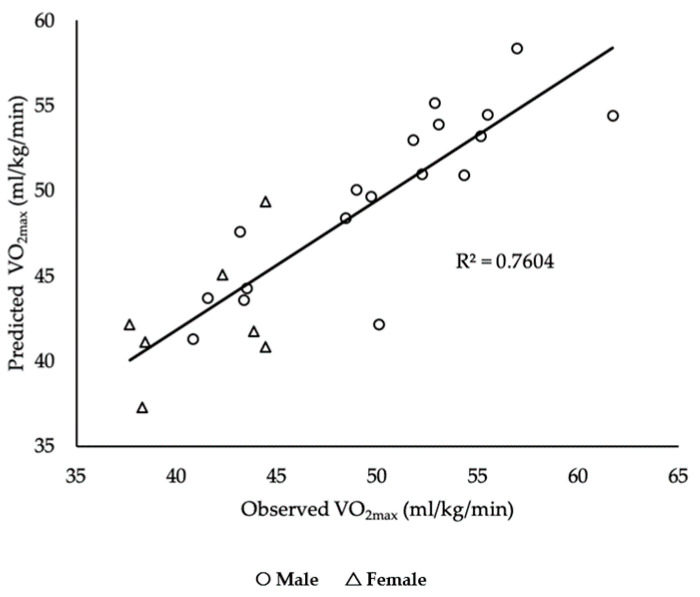
Plots of the relationship between CS and VO2_max_.

In Model 1, CS explained 67%. Model 2 added sex, which explained 76% of the variance in the model, F (1, 22) = 8.273 *p* = 0.009. Results for relative and absolute consistency are displayed in Table 2. Lastly, results from the regression analysis are displayed in Figure 1 and Table 3. 

## 4. Discussion

### 4.1. Summary 

The objective of the present study was to determine whether the 3MT may be a practical testing alternative in identifying aerobic fitness within individuals and develop a regression equation to predict VO_2max_. Significant correlations displayed between CS and relative VO_2max_ (r = 0.819, *p* < 0.001) are in line with other studies identifying statistically significant positive correlations between CS and VO_2max_ [34,35,38]. The positive correlations represent that individuals with a greater CS value also had a higher VO_2max_. Sex differences are well established, demonstrating that women may have a lower VO_2max_ compared to equally trained male counterparts due to body size and composition as well as cardiorespiratory differences [59]. Therefore, sex was included in the regression analysis. The primary finding of this study was the predictive strength of CS and sex for VO_2max_ in HIFT athletes as both CS and sex displayed significant β weights, displayed in Table 2. No significant difference was displayed between the observed VO_2max_ from the GXT and the predicted VO_2max_ from the model. High internal consistency between the values was also observed as displayed in Table 3. Findings support the use of CS, calculated from a 3MT, and sex, to predict VO_2max_.

### 4.2. Using the 3MT to Predict VO_2max_ and its Applications

To our knowledge, no previous studies have provided a regression equation for CS to predict VO_2max_, specifically using the 3MT. Moritani et al. [38] developed a regression equation to predict VO_2max_ based on cycling CP parameters determined by using the cycle ergometer in college students. The regression analysis revealed that VO_2max_ may be predicted by CP and anaerobic working capacity (AWC/W’) with a standard error of the estimate (SEE) of 0.241 L/min [38]. In rowers, Kendall et al. [34] also demonstrated CV and ARC may be used to predict VO_2max_ with a SEE of 0.161 L/min. However, it is important to note that participants in each study completed multiple trial measurements for the attainment of CP/CV and W’/ARC and not the 3MT. Moritani et al. [38] had male participants cycle at 400, 350, 300, and 275 W, while female participants cycled at 300, 250, 200, and 175 W until the onset of fatigue. Kendall et al. (2012) conducted measurements on two separate days. On day one, rowers completed distances of 400 and 1,000 m and 48 h later the rowers completed 600 and 800 m distances with 15 min between each measurement [34]. This makes for a less time efficient measurement than measuring VO_2max_ with a metabolic analyzer. The 3MT, which may also be used across multiple exercise modalities, provides measurements of CS and D’ like the multiple measurement method and has demonstrated to be a valid and reliable test for multiple modalities [30,60,61,62]. The 3MT also does not require a preliminary GXT before its implementation making it an effective alternative to assess larger numbers of athletes within a shorter span of time and decrease costs of performance testing [43].

It is important to note that in the current study, D’ did not demonstrate a significant relationship to VO_2max,_ meaning that it did not contribute to the regression model as was displayed in previous studies [34,38]. It has been proposed that W’, and presumably D’, is an indicator of anaerobic capacity displaying a significant relationship to a variety of correlates like oxygen deficit, peak blood lactate concentration or mean power during a Wingate test have been investigated and found to be significant [63,64,65]. However, these findings have also been refuted [66,67]. This may be due to the fact that there are 2 and 3 parameter linear and non-linear models as well as an exponential model to estimate W’ [68,69]. Gaesser et al. [69] examined five models for calculating W’ revealing between-model correlations from 0.25 to 0.95. Bergstrom et al. [68] further examined these five mathematical models as well as the 3MT and demonstrated the lowest W’ to come from the 3MT (10 ± 2.6 kJ) and the highest W’ from the 3-parameter non-linear model (15.2 ± 5.6 kJ). Not only is W’ variable according to the method of testing but it may also be influenced by day-to-day variations in glycogen storage [70]. Given the inconsistencies in the calculations of W’, it may be presumed that D’ would not be a good variable to include in a predictive equation. This benefits sports performance coaches and practitioners as the average speed of the last 30 s of the 3MT is the only calculation needed, simplifying the mathematical model for predicting relative VO_2max_. 

Findings suggest that CS and sex may be used to estimate VO_2max_, therefore, providing a means of assessing aerobic fitness from the 3MT. By calculating CS and estimating VO_2max_, the 3MT provides both fractional and maximal threshold values making it one of the most impactful, cost-effective, and time-efficient tests to evaluate endurance performance. As previously mentioned, numerous field-based tests exist providing an affordable means to measure fractional and maximal threshold values. For instance, the University of Montreal Track Test [71] and Cooper 12 min Run test [23] use speed and distance, respectively, to estimate VO_2max_. Several constant velocity tests, with the collection of blood lactate measurements, are used to determine fractional thresholds like LT and MLSS [24,72]. However, field-based testing methods are still time consuming and have been shown to lack accuracy, especially in athletic populations [26,73,74]. If time is a constraint, it may be argued that step tests may be used to estimate VO_2max_ [75], consequently, this this would require another test to assess a fractional threshold value. Given that VO_2max_ [6] and CS [10,40] are key indicators of endurance performance, the implementation of the 3MT to calculate both variables provides a practical means of measurement.

### 4.3. Practical Applications for Exercise Prescription

Beyond indicators of endurance performance, both CS and VO_2max_ may be used for exercise prescription. Exercise intensity is commonly prescribed a percentage of VO_2max_ (%VO_2max_). It has been proposed that %VO_2max_ should be used to prescribe exercise within the moderate domain of exercise intensity (i.e., below LT) [76] as it is less likely to elicit individual variations in blood lactate accumulation [77,78,79] which may result in too low or too high of a metabolic intensity. On the contrary, it has been noted that CS may be a more significant indicator of endurance performance than VO_2max_ [6]. The CS concept mathematically defines the relationship between speed and time to exhaustion allowing for the precise estimation of time trial completion within approximately 2.5 to 18 min [80,81]. Though these calculations are specific within the severe domain of exercise intensity, it has been proposed that taking 90% of CS may be used to predict longer time trial events [30]. Given this relationship between speed and time, the CS concept may also be used for the prescription of high-intensity interval training [82]. Further details explaining the practical application of the CS concept are presented by Pettitt [81]. Thus, the 3MT provides data that may be used to assess aerobic fitness, prescribe exercise within specific intensity domains, and predict endurance time trial performance. 

### 4.4. High-Intensity Functional Training

Findings support the use of the 3MT as a less expensive and more time efficient means of determining CS and VO_2max_, specifically in HIFT athletes. This form of training and competition emphasizes training a variety of functional movements, at a high-intensity, using minimal equipment to enhance parameters of general physical fitness [83,84]. Due to this methodology, facilities where these athlete’s train do not have access to expensive equipment necessary to evaluate and enhance key physiological performance variables, specifically VO_2max_.

HIFT has rapidly grown in popularity and has expanded to where athletes participate in competitive fitness competitions both online as well as in person at local, regional, and international events [85,86]. In these competitions, athlete’s compete in workouts that may consist of multiple exercise modalities performing workouts as fast as possible for time, for as many repetitions as possible in an allotted time, or for maximal weight lifted. A large variety of physiological factors have previously been identified that influence HIFT athlete’s performance, with VO_2max_ being a significant predictor of competitive success [41,87,88,89,90,91,92]. Bellar et al. [90] identified VO_2max_ to be a significant indicator of workout performance by revealing a positive and significant correlation between maximum aerobic capacity and the amount of repetitions performed in a timed workout. This was further supported by findings in our laboratory [41] as well as Martinez-Gomez et al. [93] who also demonstrated VO_2max_ to be a significant indicator of HIFT workout performance. However, it is important to note that VO_2max_ as an indicator of HIFT workout performance may be mode specific. Maximal oxygen uptake was previously assessed on a longer HIFT workout. However, the workout did not consist of the mode that the GXT was conducted on, which was on a cycle ergometer; the workout consisted of push-ups, pull-ups, and air squats for as many repetitions as possible in 20 min [89]. Thus, it is important to emphasize specificity in testing and workout modalities. The significant relationship of VO_2max_ to HIFT performance is an important reason to implement a 3MT for the prediction of VO_2max_. This does not mean that CS may not also play a role in HIFT workout performance. Future research should also investigate CS as a potential indicator of HIFT workout performance as critical power (CP) measured from a 3MT cycling test revealed that more competitive HIFT athletes have higher a higher CP [87]. Lastly, both VO_2max_ and CS have previously demonstrated a significant relationship to HIFT workout performance on the same workout; however, VO_2max_ significantly predicted performance [41]. 

### 4.5. Limitations and Future Research

This study is not without its limitations. Future research should investigate the use of this equation on a HIFT recreationally athletic population. Kendall et al. [34] assessed participants not used in the formation of the regression equation and determined the SEE to be 0.144 L/min. This study did not include a separate validation sample outside of the subjects used to develop the equation, therefore, future validation studies are warranted. Both males and females were used in the conception of the regression equation and sex significantly contributed to the model. The present study had a lower number of females and the sample size as a whole is small for building a regression equation. However, a post-hoc power analysis as well as low *p*-values and a high r-squared value indicate high statistical power and a strong prediction model. 

## 5. Conclusions

In conclusion, the results of this study support the use of the 3MT as a practical field test for predicting VO_2max_ in recreational HIFT athletes. The 3MT is a non-invasive field test and may contribute as a time effective alternative in which larger numbers of athletes can complete the procedure within a shorter span of time as compared to the time-intensive measuring of VO_2max_ via a GXT. Future investigations should evaluate the validity of this equation as well as examine men and women separately due to physiological sex differences. The field-based method of the 3MT, along with the generated VO_2max_ prediction equation, would allow coaches and training professionals to accurately predict VO_2max_ in athletes while affording a practical method to predict, monitor, and refine performance in a timely manner.

## Figures and Tables

**Table 1:** Participant Characteristics.

	Total	Males	Females
N Age (years)	25 27.6 ± 4.5	17 27.6 ± 5.1	8 27.5 ± 3.3
Height (cm)	174.5 ± 18.3	178.8 ± 20.3	165.4 ± 8.6
Weight (kg)	77.4 ± 14.8	83.5 ± 12.9	64.5 ± 9.1
Body Fat (%)	15.7 ± 6.5	13.3 ± 6.4	21.1 ± 2.6

Note: The values are express as the mean ± standard deviation (SD).

**Table 2:** VO_2max_ prediction equation measures of reliability Model 2.

Observed VO_2max_ (ml/kg/min^−1^) †	Predicted VO_2max_ (ml/kg/min^−1^) †	TE (mL/kg/min^−1^)	%CV	ICC	Lower 95% CI (ml/kg/min^−1^)	Upper 95% CI (ml/kg/min^−1^)
47.71 ± 6.54 ^a^	47.71 ± 5.70 ^a^	2.26	4.90	0.864	1.77	3.15

^a^ Not significantly different (t = −0.000, *p* = 1.000). Abbreviations: TE = typical error; %CV = coefficient of variation; ICC = intraclass correlation coefficient; CI = confidence interval. † Values are given as the mean ± SD.

**Table 3:** Summary of Regression Analysis Model 2.

Variable	B	SE_B_	β	Observed Power
CS (m/s)	8.449	1.323	0.709 **	0.99
Sex (M/F)	4.387	1.525	0.320 **	

Note: ** *p* < 0.01; B = unstandardized regression coefficient; SE_B_ = standard error of the coefficient; β = standardized coefficient; observed power = post-hoc power analysis.
